# Joint-preserving palliative surgery using self-locking screws of intramedullary nail and percutaneous cementoplasty for proximal humeral metastasis in the advanced cancer patients

**DOI:** 10.1186/s12957-018-1397-3

**Published:** 2018-05-15

**Authors:** Jong Woong Park, Yong-il Kim, Hyun Guy Kang, June Hyuk Kim, Han Soo Kim

**Affiliations:** 1 Orthopaedic Oncology Clinic, National Cancer Center, 323 Ilsan-ro, Ilsandong-gu, Goyang-si, Gyeonggi-do 10408 Republic of Korea; 2 Department of Nuclear Medicine, Asan Medical Center, University of Ulsan College of Medicine, Seoul, Republic of Korea; 3 Department of Orthopaedic Surgery, Seoul National University Hospital, Seoul, Republic of Korea

**Keywords:** Self-locking screw, Percutaneous cementoplasty, Bone metastasis

## Abstract

**Background:**

We introduced a palliative joint-preserving surgery using proximal self-locking screws of intramedullary (IM) nail and percutaneous cementoplasty (PC) in patients with proximal humeral metastases, including the head and neck, and evaluated the outcome of the surgical method.

**Methods:**

Twenty-three patients (mean age = 63.0 ± 11.8 years, M:F = 14:9) had IM nailing with a self-locking screw system and PC for the treatment of humeral head and neck metastases. Usually, three proximal locking screws were inserted after IM nailing, and 20.9 ± 8.0 ml of polymethylmethacrylate (PMMA) bone cement was injected in the perimetal osteolytic area.

**Results:**

Regional anesthesia with interscalene block was performed in 87.0% (20/23), and the duration of surgery (from anesthesia to awakening) was approximately 40–55 min. Red blood cell was not transfused intra- and/or postoperatively in 65.2% (15/23). The localized preoperative pain (visual analog scale (VAS), 8.2 ± 3.1) was gradually decreased at postoperative 1 week (VAS, 4.9 ± 2.1) and at 6 weeks (VAS, 2.9 ± 2.1) (*P* < 0.001). Among nine patients who underwent F-18-FDG PET/CT, the proximal humeral metastasis around PC showed improved, stable, and aggravated states in five (55.6%), three (33.3%), and one patient (11.1%), respectively. Meanwhile, 88.8% (8/9) of patients showed aggravation at the naive bone metastasis area.

**Conclusion:**

The selection of the self-locking screw type of the IM nail and PC was helpful in preventing fixation failure for joint-preserving palliative surgery in the proximal humeral metastasis.

## Background

The management of humerus metastasis is to alleviate patients’ symptoms and maintain function, and surgical approach has benefits by reducing local pain and early mobilization [1, 2]. Intramedullary (IM) nail fixation is the main treatment for long bone and humeral metastases [3–5]. However, obtaining a successful result for the proximal humeral metastasis is difficult, because the possibility of fixation failure is high in IM nail fixation, and the risk of open surgery and tumor progression is high in plate fixation. Hence, joint replacement surgery after marginal or wide excision was mainly performed in the proximal part of the humerus [6].

The self-locking screw type of the IM nail had been introduced for surgical treatment of proximal femoral fracture. The gamma nail has the locking mechanism between an IM nail and screws, which lowers the possibility of loosening in osteoporotic bones [7]. For the proximal humerus, the self-locking screw type of the IM nail (Trigen Humeral Nail; Smith and Nephew, USA) has been introduced to inhibit loosening failure of osteoporotic bone [8]. There is a unique thread inside the hole to make a locking mechanism between the IM nail and proximal screws. Theoretically, when osteolysis progresses postoperatively, loosening would not occur in the proximal part of the humerus due to the self-locking mechanism between the IM nail and screws.

The percutaneous cementoplasty (PC) of polymethylmethacrylate (PMMA) is a useful method for metastatic bone disease [9, 10]. By PC, patients achieved good pain relief and early function recovery of the extremities [10, 11]. However, PC alone is not sufficient for maintaining long bone strength against pathologic fracture [12]. Recently, PC is combined with flexible or rigid IM nail for long bone metastasis [9, 13, 14] or hollow perforated screws for femur neck metastasis [15, 16] and showed durable stability and effective local tumor suppression.

We hypothesized that proximal self-locking screw insertion of an IM nail and combination with PC can be useful for salvaging the shoulder joint in the humeral head and neck metastasis as a palliative minimally invasive surgery.

## Methods

### Patients

Among 122 patients who underwent humeral metastasis surgery between July 2012 and May 2015, 98 patients whose metastatic area was the proximal humerus were retrospectively reviewed. In the proximal humerus, surgical treatment for bone metastasis was performed mainly in patients with impending/pathologic fractures by osteolytic bone metastasis. For patients with osteoblastic bone metastasis, surgical treatments were performed by other methods than self-locking type IM nailing, because mechanical failure risk was expected low. Among patients who underwent surgical treatment for the proximal humerus metastasis, 23 patients underwent rigid IM nailing with the self-locking screw type to stabilize the proximal humerus. In our institute, PMMA was routinely used for osteolytic long bone metastasis after metal fixation since 2008. For 23 patients in final analysis, all patients had osteolytic bone metastasis in the proximal humerus and PC was used as combined treatment.

All patients met the criteria of the Mirel score of > 9 for humeral metastasis [17]. The primary malignancies were the lungs, kidney, liver, breast, prostate, multiple myeloma, lymphoma, and salivary gland in eight patients, four patients, three patients, two patients, two patients, two patients, one patient, and one patient, respectively.

### Surgical procedures

**Fig. 1:**
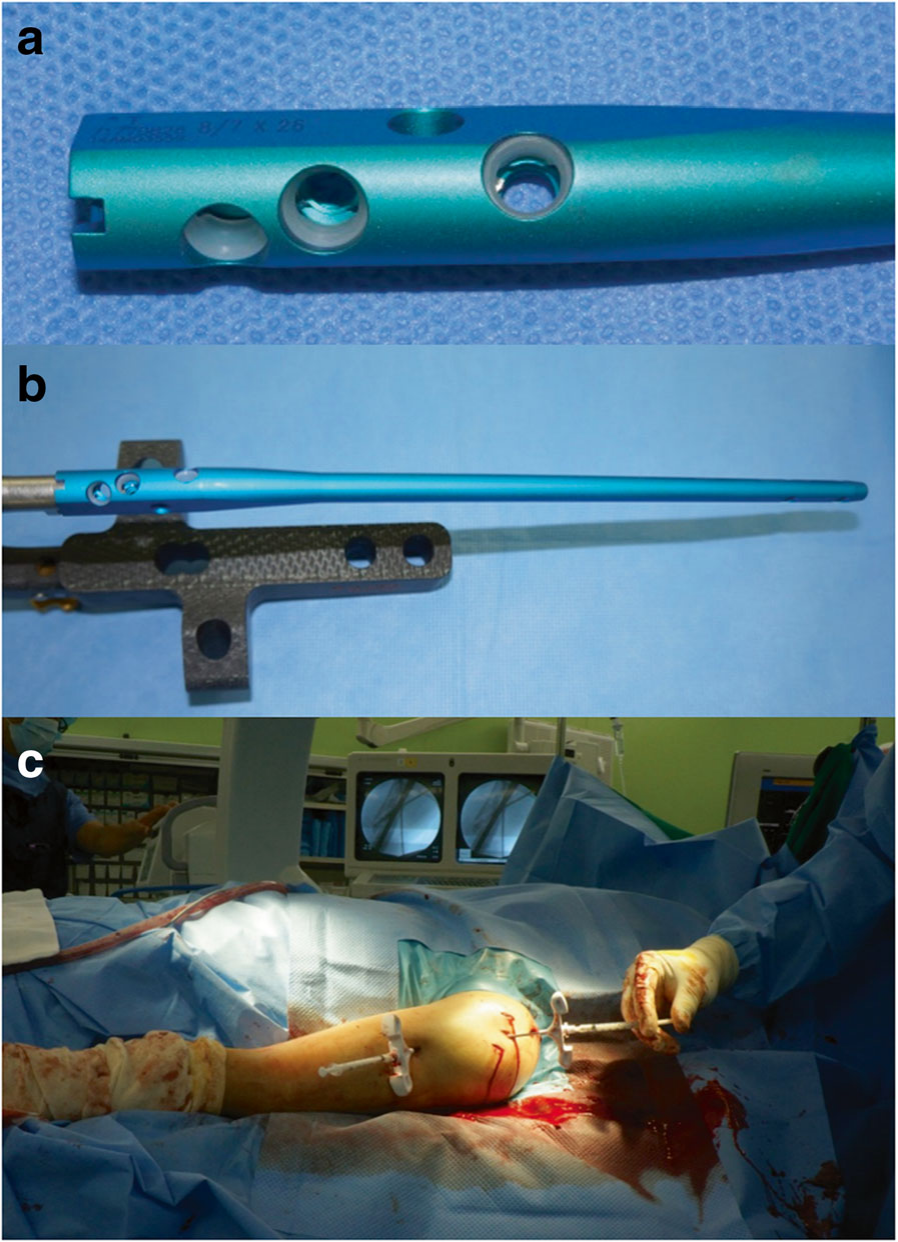
The proximal magnified image (**a**) and overview (**b**) of self-locking screw type IM nail, which prevents loosening of the screws. The photograph shows the PC (**c**)

All patients were positioned supine with head slightly up. We used the deltoid-splitting approach, and the supraspinatus tendon was divided sharply using a knife for easy repair after nail insertion. The entry point was created just medial to the supraspinatus tendon insertion in the anteroposterior view and was centered in the lateral view. We selected the smallest diameter with measured length of the nail and assembled it before proximal reaming. Through the entry hole in the proximal humerus, the self-locking screw type of the humeral IM nail (Trigen Humeral Nail; Smith and Nephew, USA) was inserted according to the usual proximal humeral IM nail method. Three 4.5-mm proximal locking screws were inserted using a targeting device. Distal locking screw fixation was optional. After targeting device removal, fluoroscopic check of the nail insertion and fixation of the proximal self-locking screws was performed (Fig. 1a, b).

For PC, a percutaneous vertebroplasty (PV) needle (10 gauge, 11 cm; poverty needle; Kyungwon Medical, Seoul, Korea) was inserted into the medullary cavity by hand-pushing or hammering in a percutaneous and transcortical manner. Additional PV needles were inserted in cases of long intramedullary or skipped lesions. The entry point of the needle was selected at the most easily accessible area to the lesion, which was apart from the neurovascular bundles. After identifying the location of the PV needle by fluoroscopy, the low-viscosity radiopaque PMMA (Depuy International Ltd., Blackpool, UK) was mixed for 2 min and transferred to a 30- or 50-ml syringe, depending on the number of cement pack (20 g/pack). Subsequently, PMMA was transferred again into 18 to 20 1-ml syringes/pack and injected 3–4 min after mixing. The initial PMMA was slowly injected by checking venous drainage by monitoring using fluoroscopy and evaluating blood pressure. Bone cement was injected as much as possible, with frequent checking of the anesthetic monitor and fluoroscopy (Fig. 1c). The PV needle was removed before complete cement solidification, which was approximately 10–12 min after mixing.

### Additional therapy

The conventional treatment for bone metastasis, radiation therapy, and/or chemotherapy was decided by a multidisciplinary team approach of the hemato-oncologists, radiation oncologists, and orthopedic surgeons [18]. The patients who were indicated adjuvant therapy were referred for adjuvant radiation therapy and/or chemotherapy without delay postoperatively because only stab wounds were made with our method.

### Self-locking screw insertion of IM nail with PC result evaluation

The patients’ anesthesia method, duration of surgery, red blood cell (RBC) transfusion, pain killer use, and complication were checked by reviewing the medical records. In addition, each patient was asked to quantify pain based on the numeric visual analog scale (VAS) with values from 0 (absence of pain) to 10 (strongest pain ever experienced) a day before surgery and 1 and 6 weeks postoperatively [19]. Postoperative bone destruction status was evaluated by plain radiography.

### F-18-FDG PET/CT image acquisition and evaluation

To evaluate the patients’ tumor progression status, F-18-FDG PET/CT was evaluated in patients who performed before and after the self-locking screw insertion with PC. PET/CT was taken when needed from a multidisciplinary point of view for systemic assessment, and no case was taken for the purpose of tracking only bone metastasis. There were nine patients who had both preoperative and postoperative PET/CT. We evaluated the progressiveness of bone metastases in each individual patient between the proximal self-locking screw insertion of IM nail with PC area and other naive bone metastasis areas. PET/CT evaluation was grouped into three categories: improved, stable, and aggravated states. The improved state was defined as decreased extent and intensity of lesion uptake, the stable state was defined as no significant interval change, and the aggravated state was defined as increased extent and intensity of uptake or new metastatic lesion. In addition, the maximum standardized uptake value (SUVmax) calculated was acquired based on the following equation to quantify F-18-FDG uptake: SUV = (tissue radioactivity [Bq/ml])/(total injected activity [Bq]/body weight [g]).

### Statistical analysis

The patients’ pain score changes (preoperative vs. postoperative 1 week vs. postoperative 6 weeks) were evaluated by repeated measures of analysis of variance (ANOVA). A *P* value < 0.05 was considered significant. The statistical analyses were performed using the software package SPSS 18.0 (IBM, Chicago, IL, USA).

## Results

### Patients’ baseline characteristics

Our study included 23 patients (mean age = 63.0 ± 11.8 years, range = 36–80 year; M:F = 14:9). The Mirel scores were 9 and 10 in 7 (30.4%) and 16 patients (69.6%), respectively. Radiation therapy and chemotherapy for the proximal humeral metastases were performed in 91.3% (21/23) and 95.7% (22/23) of the patients, respectively. The mean follow-up duration was 6.3 ± 6.2 months (Table 1).

**Table 1:** Patients’ demographics

Patient characteristics (*n* = 23)	Values
Age (mean ± SD, years)	63.0 ± 11.8 (range, 36–80)
Gender (number, M:F)	14:9
Follow-up duration (mean ± SD, months)	6.3 ± 6.2 (range 0.4–22.6)
Mirel’s score	
Score 9	7
Score 10	16
Pre-op. pain score (visual analogue scale (VAS))	8.2 ± 3.1 (range, 2–10)
Combined radiation therapy (pre- and/or post-op.)	21 (91.3%)
Combined chemotherapy (pre- and/or post-op.)	22 (95.7%)

*Op.* operative

### Clinical results of self-locking screw insertion with PC

**Fig. 2:**
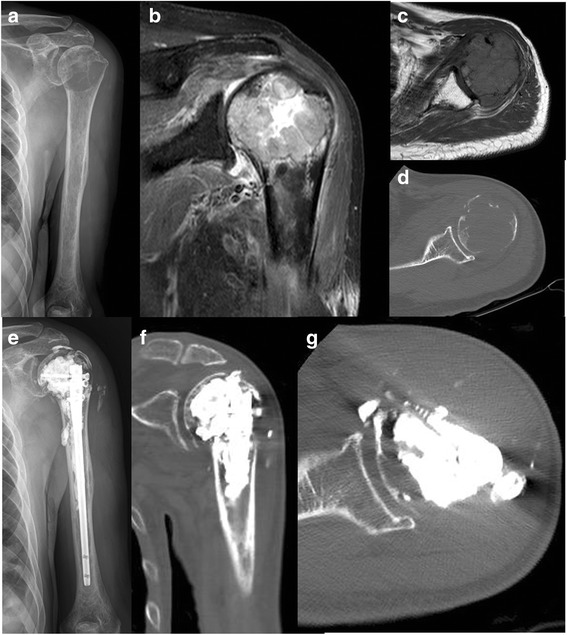
A case of a 62-year-old man with multiple lung, brain, and bone metastases due to advanced renal cell carcinoma. Preoperative plain radiograph (**a**), fat-suppressed enhanced coronal MRI (**b**), T1-weighted axial MRI (**c**), and CT (**d**) show osteolytic lesion in the humeral head. Postoperative evaluation reveals stable fixation by proximal self-locking screws of IM nail and PC in plain radiograph (**e**), coronal reconstructive CT (**f**), and axial CT (**g**)

**Fig. 3:**
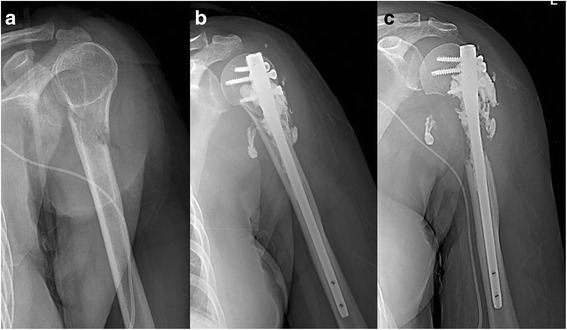
A case of a 56-year-old woman with multiple bone and liver metastases due to advanced breast cancer. Pathologic fracture of the humeral neck (**a**) is fixed with a self-locking screw type of IM nail and PC (**b**). The progressive osteolysis to the humeral head is limited and no fixation failure occurred during follow-up period (**c**)

**Fig. 4:**
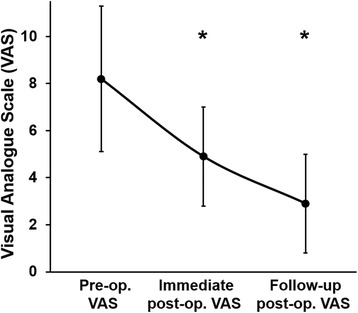
Visual analog scale (VAS) score changes after insertion of self-locking screws with PC. The VAS of the patients was 8.2 ± 3.1 preoperatively. The patients’ VAS decreased significantly to 4.9 ± 2.1 1 week postoperatively (immediate postop VAS) (*P* < 0.001). The patients’ VAS further decreased significantly to 2.9 ± 2.1 6 weeks postoperatively (follow-up postop VAS) (*P* < 0.001)

Three proximal self-locking screw insertions and PC were successfully performed in all patients without major complication. The patients underwent regional and general anesthesia in 87.0% (20/23) and 13.0% (3/23), respectively. The injected cement amount was 20.9 ± 8.0 ml (range = 11–40 ml). The mean duration of surgery was approximately 40 min without distal locking screw insertion (with insertion = 55 min), and two packs (400 ml/pack) of RBC was transfused in 34.8% (8/23) of patients (no RBC transfusion in the remaining 15 patients). Distal locking screws were not inserted in nine patients (with insertion = 14 patients). Preoperative embolization in the nail insertion track was not performed in any of the patients. Postoperatively, good immediate structural stability was achieved in all patients (Fig. 2), and durability was achieved even when the adjacent bony structure was severely damaged (Fig. 3). Postoperative complication was merely transient delirium in 13.0% (3/23) of patients. At the final follow-up, one patient survived, 16 patients died of disease, and six patients were lost to follow-up (Table 2). The patients’ preoperative VAS was 8.2 ± 3.1. The patients’ pain was gradually decreased 1 week (VAS, 4.9 ± 2.1) and 6 weeks postoperatively (VAS, 2.9 ± 2.1) (*P* < 0.001; Fig. 4).

**Table 2:** Results of self-locking screw insertion with PC

Number	Age	Gender	Primary tumor	Anesthesia	Injected cement amount (ml)	RBC transfusion (pint)	Post-op. bone destruction	Post-op. complication	Status
1	72	M	Lung	General	20	No	Destruction	Delirium	DOD
2	73	F	Lung	General	18	2	None	No	DOD
3	63	M	Liver	General	19	No	Destruction	No	DOD
4	75	F	Multiple myeloma	Regional	11	2	None	No	DOD
5	72	M	Lymphoma	Regional	20	2	Destruction	No	DOD
6	72	M	Kidney	Regional	18	2	None	No	AWD
7	48	F	Salivary gland	Regional	13	No	None	No	DOD
8	55	F	Kidney	Regional	17	2	Destruction	No	DOD
9	54	M	Kidney	Regional	26	2	Destruction	No	DOD
10	52	F	Breast	Regional	21	No	Destruction	No	FU loss
11	73	M	Liver	Regional	19	No	None	No	FU loss
12	63	F	Liver	Regional	18	2	None	No	DOD
13	36	M	Lung	Regional	22	No	Destruction	No	FU loss
14	65	F	Multiple myeloma	Regional	12	No	None	Delirium	FU loss
15	75	M	Prostate	Regional	16	No	Destruction	No	DOD
16	71	M	Lung	Regional	30	No	None	No	FU loss
17	40	M	Lung	Regional	40	No	None	Delirium	FU loss
18	80	M	Lung	Regional	40	No	None	No	DOD
19	67	M	Lung	Regional	16	No	None	No	DOD
20	54	F	Lung	Regional	12	2	Destruction	No	DOD
21	56	M	Kidney	Regional	20	No	None	No	DOD
22	61	M	Prostate	Regional	33	No	None	No	DOD
23	71	F	Breast	Regional	20	No	None	No	DOD

*PC* percutaneous cementoplasty, *RBC* red blood cell, *DOD* died of disease, *AWD* alive with disease, *FU* follow-up

### Bone destruction and tumor progression evaluation

Postoperative progressive bone destruction of the proximal humerus was found in 39.1% (9/23) of patients by simple pain radiography; however, the patient showed no screw back-out and fixation failure.

**Fig. 5:**
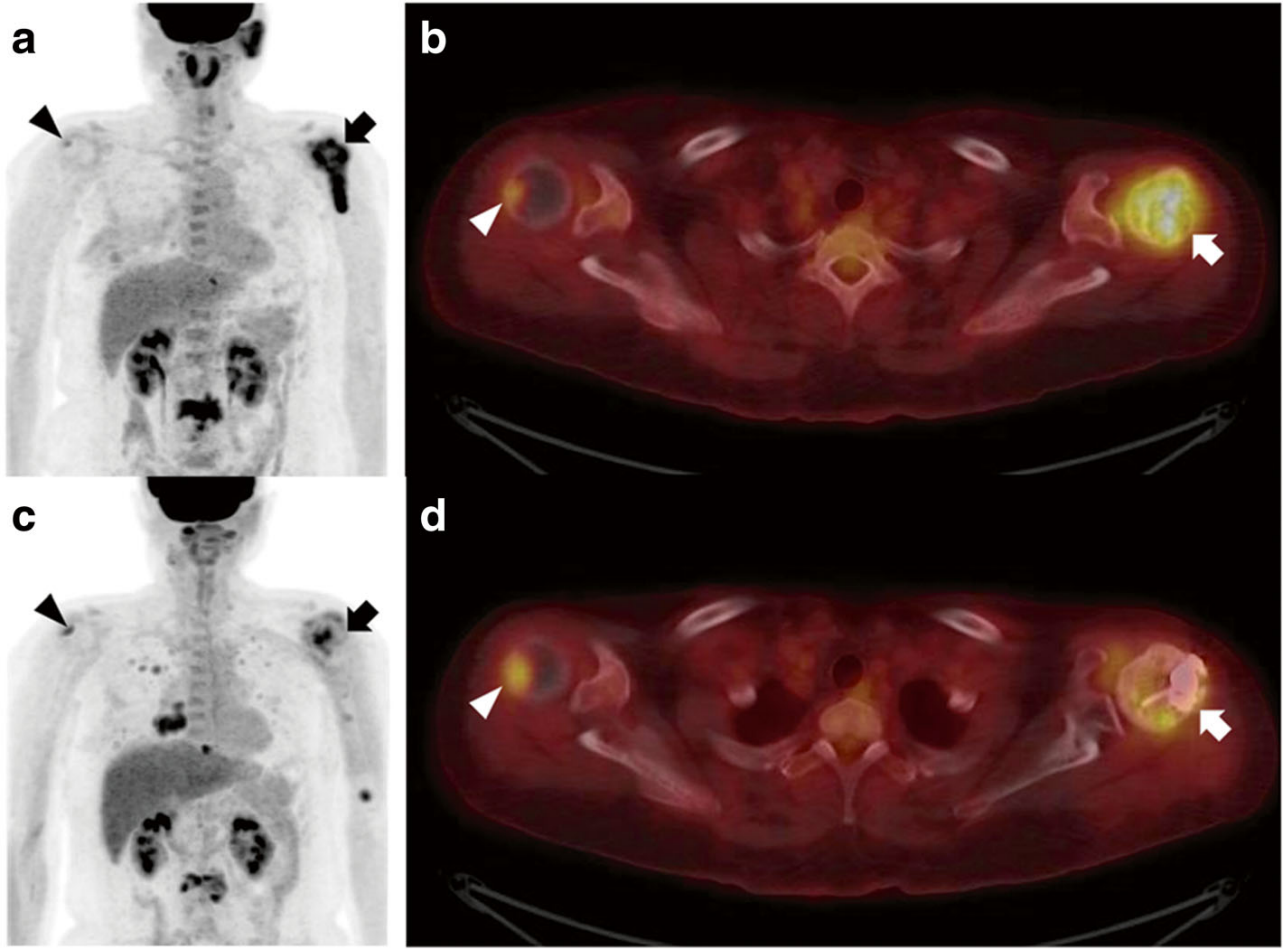
Suppression of tumor progression by self-locking screws with PC. A 48-year-old woman with multiple bone metastases due to salivary gland tumor. Preoperative maximal intensity projection (MIP) (**a**) and transaxial (**b**) images of F-18-FDG PET/CT show hypermetabolic lesions in the left (arrow; maximum standardized uptake value [SUVmax] = 11.4) and right (arrowhead; SUVmax = 2.8) proximal humerus. Six months postoperatively, the MIP (**c**) and transaxial (**d**) images of F-18-FDG PET/CT demonstrated tumor suppression of the right proximal humus, the op. site (arrow; SUVmax = 5.5). However, naive metastatic area left proximal humerus (arrowheads; SUVmax = 3.8) reveal tumor aggravation of bone metastasis, and multiple new metastatic lesions were found in the lungs and T10 and L3 spines

Of 23 patients, there were nine patients who were finally included for the evaluation of F-18-FDG PET/CT study. Preoperative F-18-FDG PET/CT was performed 1.3 ± 0.6 months preoperatively, and postoperative F-18-FDG PET/CT was performed 2.7 ± 0.8 months postoperatively. Among them, the proximal humeral metastasis around PC showed improved, stable, and aggravated states in five (55.6%), three (33.3%), and one patient (11.1%), respectively. SUVmax change in self-locking screws with PC area was from 9.1 ± 3.0 to 7.6 ± 2.5. In contrast, the naive bone metastasis area demonstrated aggravated state in eight patients (88.9%) and improved state in one patient (11.1%). SUVmax changes of naive bone metastasis area were from 2.5 ± 0.8 to 6.1 ± 2.0 (Table 3 and Fig. 5).

**Table 3:** F-18-FDG PET/CT results after self-locking screw insertion with PC

Number	Evaluation of self-locking screws with PC area	Pre-op. SUVmax (op. site)	Post-op. SUVmax (op. site)	Evaluation of naive bone metastasis area	Pre-op. SUVmax (naive bone metastasis)	Post-op. SUVmax (naive bone metastasis)
1	Stable	4.38	4.27	Aggravated (T9 spine)	6.69	9.73
2	Improved	6.46	2.64	Aggravated (pleura)	1.56	5.78
3	Improved	6.76	3.58	Aggravated (sternum)	1.71	6.12
4	Improved	31.92	26.71	Aggravated (right clavicle)	8.66	21.95
5	Improved	7.48	3.19	Improved (left femur)	5.19	2.36
6	Stable	3.34	3.97	Aggravated (right lung)	2.19	2.69
7	Improved	11.42	5.51	Aggravated (right humerus)	2.80	3.84
8	Aggravated	3.48	7.36	Aggravated (right 8th rib)	3.39	6.17
9	Stable	3.30	4.03	Aggravated (L2 spine)	2.68	3.94

*PET* positron emission tomography, *CT* computed tomography, *SUVmax* maximum standardized uptake value

## Discussion

The proximal self-locking screws of IM nail were helpful to maintain stability without loosening in severely damaged bone by metastasis. Furthermore, combined PC not only contributed to maintaining stability, but also helped suppress bone metastasis. In our study, no loosening and fixation failure occurred even in extensive osteolysis by self-locking screw insertion and PC.

Pain relief and pathological fracture prevention are important when treating patients with metastatic bone cancer. Closed intramedullary nailing is the most useful method for metastatic cancer of the long bone in the limbs [20]. The IM nail with self-locked screws were initially introduced in proximal femur fractures and showed pain relief, function recovery, and decreased reoperation rate [21]. Additional stability that was achieved by the locking mechanism between screws and an IM nail may also be useful for pathological fractures in long bones. However, because closed intramedullary nailing in the long bone does not remove tumor tissue, patients are at risk for progressive bone destruction due to intramedullary spreading of the tumor during medullary reaming or inserting of the nail. In order to overcome the disadvantages of closed intramedullary nailing, using a nail with the smallest diameter had some advantages when treating long bone metastasis to avoid reaming and pushing down the tumor to the distal marrow space. In addition, when PC is combined, a nail with the smallest diameter provides a sufficient space between the nail and cortical bone for injecting bone cement. Unlike the femur, a small-sized unreaming nail (8 mm) may be sufficient because the humerus is a non-weight-bearing bone. In this study, there were no patients who encountered mechanical failure in the proximal humerus. The distal locking screws were inserted in the patients with pathologic fractures of the distal one third of the proximal humerus or combined bone metastasis in the distal one third of the humerus. Nine patients (39.1%) had no distal locking screws, and the duration of surgery and radiation exposure by intraoperative fluoroscopy were reduced in these patients.

Combining PC while performing proximal self-locking screws of IM nail insertion has many advantages. First, intraoperative bone bleeding was stopped by bone cement injection, and postoperative bleeding was also reduced. Preoperative embolization procedure was not necessary even in bone metastasis of renal cell carcinoma which is a well-known hypervascular tumor. Second, because the bone cement fills the microfracture and the gap between an osteolytic bone and the nail, immediate fixation stability is improved, resulting in immediate pain relief. Third, combined PC influences local tumor suppression by inhibiting intramedullary tumor spreading. When PC was performed in the intramedullary area, the cement was distributed around the normal intramedullary area as well as osteolytic lesion [13]. The blocking effect of nutrition and cytotoxic effect of the tumor was induced by cement and achieved tumor volume reduction and tumor necrosis [22]. Because F-18-FDG PET/CT is a good imaging modality to detect bone metastasis [23], it can be used to identify the tumor suppression effect of the treatment [14]. In the study, aggravation of metastasis around self-locking screws with the PC was found in only one case (11.1%), which implies effective tumor control. In contrast, other naive metastasis area showed increased uptake in most cases (88.9%), which means ineffective tumor control. The most worrisome complications during bone cement injection are fat and cement embolisms [24, 25]. In our study, no major complications, including fat or bone cement embolisms, occurred.

Our study has some limitations. The number of the patients was relatively small. In addition, because the patients had multiple factors associated with patients’ status, VAS pain score was difficult to evaluate at follow-up. Finally, radiation therapy and/or chemotherapy factors could be the confounding factor for exact evaluation. The influence of combined therapy and comparison with standard surgical technique should be performed in the future.

## Conclusions

The self-locking screw insertion of IM nail with PC is a feasible procedure as a minimally invasive and joint-preserving surgery for proximal humeral metastasis of patients with advanced cancer.

## Abbreviations

IM: Intramedullary
PC: Percutaneous cementoplasty
PMMA: Polymethylmethacrylate
